# Supplementary material for: Hypothermic Protection in Neocortex Is Topographic and Laminar, Seizure Unmitigating, and Partially Rescues Neurons Depleted of RNA Splicing Protein Rbfox3/NeuN in Neonatal Hypoxic-Ischemic Male Piglets
Source: Cells. 2023 Oct 15;12(20):2454. doi: 10.3390/cells12202454 (PMC10605428; doi:10.3390/cells12202454)
Supplement: Supplementary file 1 [file cells-12-02454-s001.zip › cells-2616696-supplementary.pdf]

Hypothermic Protection in Neocortex Is Topographic and

Laminar, Seizure Unmitigating, and Partially Rescues Neurons Depleted of RNA Splicing Protein Rbfox3/NeuN in Neonatal

Hypoxic-Ischemic Male Piglets

Supplementary Materials:

Supplemental Videos: https://zenodo.org/record/8106022

Animal attrition and interventions

Forty piglets were anesthetized for intubation and catheter placement. Among piglets randomized to HI and NT, one animal died of refractory hypotension during anesthesia and a second piglet could not be extubated. One HI-HT piglet had a pulmonary hemorrhage, and another had a cardiac arrest, excluding them from the study. One sham-HT piglet could not be intubated and a second did not sustain adequate ventilation upon extubation and thus did not complete the protocol. Three HI-HT, 1 HI-NT, and 1 sham-HT piglet received dopamine during anesthesia.

Anesthesia toxicity

To examine the potential effects of the anesthetic regimen with and without HT, we compared the ratio of normal-to-total neurons among naïve unanesthetized (n=6), sham NT (n=6), and sham HT (n=10) piglets. The ratio of normal neurons did not differ between anterior and mid-parietal somatosensory and motor cortices among the unanesthetized and sham procedure piglets ($p>0.05$ for all comparisons).

Supplementary Figure S1

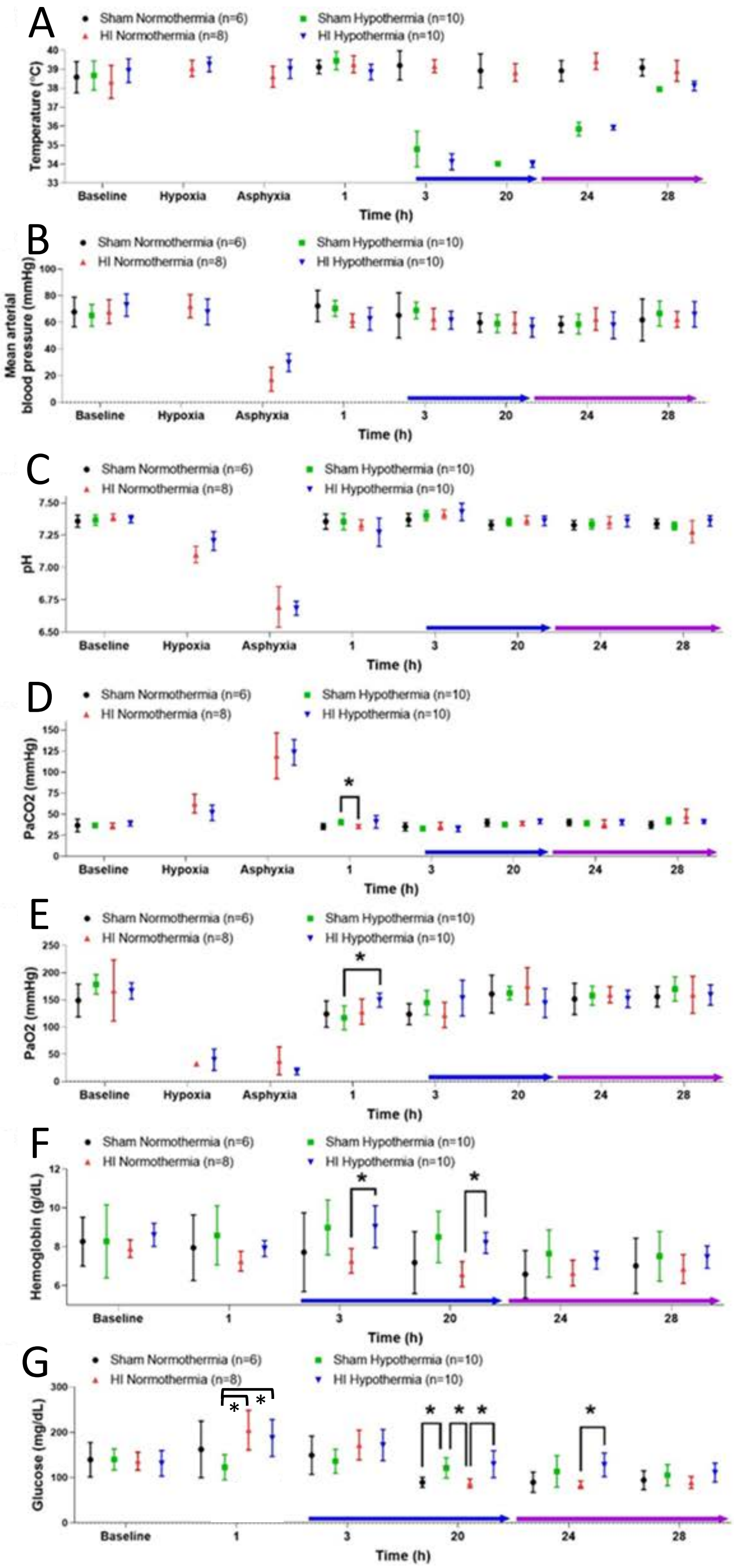

Supplementary Figure S1 Legend. Physiology during piglet insult protocol including during anesthesia. Components of this figure are shown in Figure 6 of the main paper. The blue arrows along the x-axis show the beginning and maintenance of hypothermia. The purple arrows show rewarming. Piglets' core temperatures **(A)**, mean arterial blood pressure **(B)**, and arterial pH **(C)** are shown. Time and treatment interactively affected the arterial partial pressure of carbon dioxide ($PaCO_2$; p=0.006; **D**). The arterial partial pressure of oxygen (PaO_2) (p<0.001; **E**) and hemoglobin levels also varied across time (p<0.001; **F**). Glucose was interactively affected by time and treatment (p<0.001; **G**). *p<0.05 in post-hoc tests. Data are shown as means with 95% confidence intervals. Blood gas, hemoglobin, and glucose data are from 7-min asphyxia. Temperature and blood pressure data are from 8-min asphyxia.

Supplementary Figure S2.

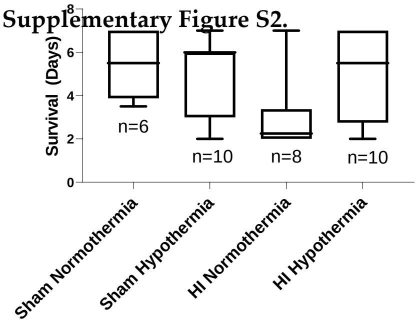

Supplementary Figure S2 Legend. Piglet treatment group survival.The duration of survival did not differ

among groups (p=0.078).

Supplementary Figure S3

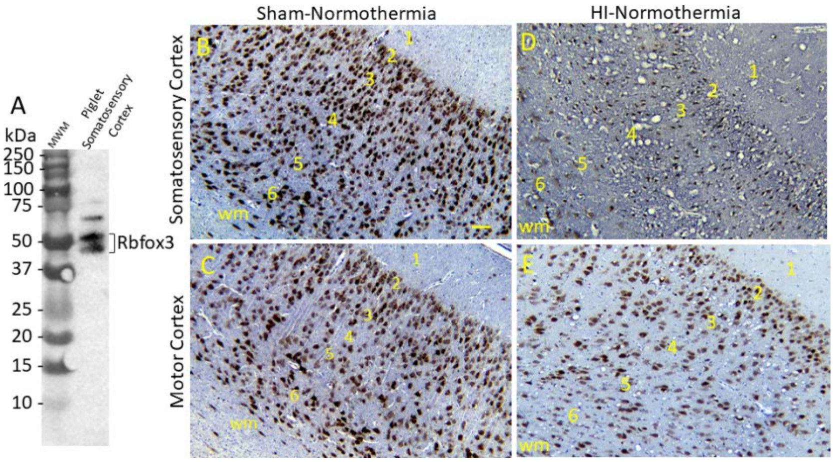

Supplementary Figure S3 Legend. Rbfox3/NeuN in piglet cerebral cortex. Panels A, B, and C of this figure are shown in Figure 3 in the main paper. A. Larger size image of western blot shown in Figure 3A inset. The antibody is highly specific. Panels B and D are coronal section magnification- and orientation-matched images of RBFOX3/ NeuN immunohistochemical staining in the somatosensory cortex of sham-normothermia and HI-normothermia piglets. The numbers identify the cortical layers from layer 1 to the subcortical white matter (wm). The H piglet has marked low of RBFOX3/NeuN staining in later-specific patterns so that layer identification is still possible. Scale bar in B = 70

μm (same for C,D,E). Panels C and E are coronal section magnification- and orientation-matched images of RBFOX3/NeuN immunohistochemical staining in the motor cortex of sham-normothermia and HI-normothermia piglets. The motor cortex was vulnerable in HI piglets but was less vulnerable in some piglets with less severe seizures. There is loss of positive neurons particularly in layer 3.

Supplementary Figure S4

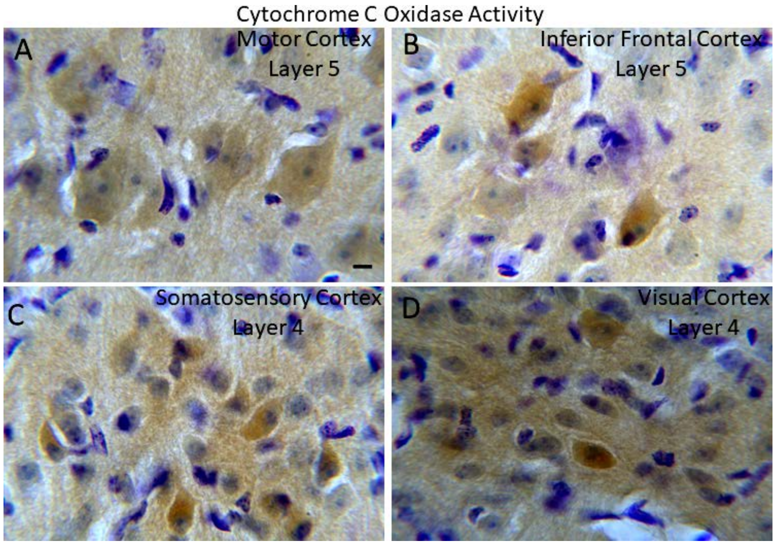

Supplementary Figure S4 Legend. Cytochrome C oxidase enzyme activity in different neocortical layers of piglet. This figure complements Figure 4E-G in the main paper by showing high magnification images of the localization of the cytochrome c oxidase enzyme activity in specific layers of motor cortex (A), orbitofrontal cortex (B), primary somatosensory cortex (C), and visual cortex of naïve piglet. Scale bar in A = 10 μm (same for B-D).

Supplementary Figure S5

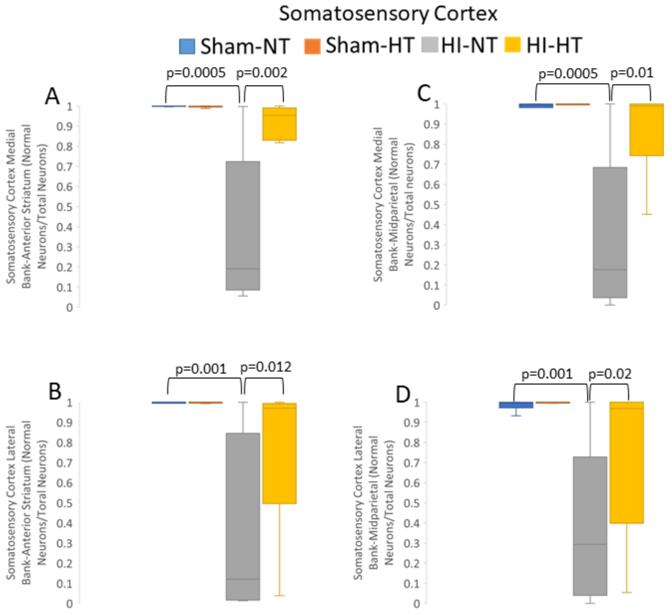

Supplementary Figure S5 Legend. Topographically different regions of primary somatosensory neocortex were protected in HI-HT piglets: normal neuron-to-total neuron ratios. **A,B.** Anterior somatosensory cortex at an anterior striatal level. **C,D.** Posterior somatosensory cortex at a midparietal/thalamus level. Compared to sham-NT piglets, HI-NT piglets had decreased normal-to-total neuron ratios in the somatosensory gyrus in anterior parietal cortex medial bank (*p*<0.001) and lateral bank (p=0.001) and in the mid-parietal cortex (medial: *p*<0.001; lateral: *p*=0.001). These neocortical regions in HI-HT piglets had significant rescue from neuron loss compared to NI-NT

Supplementary Figure S6

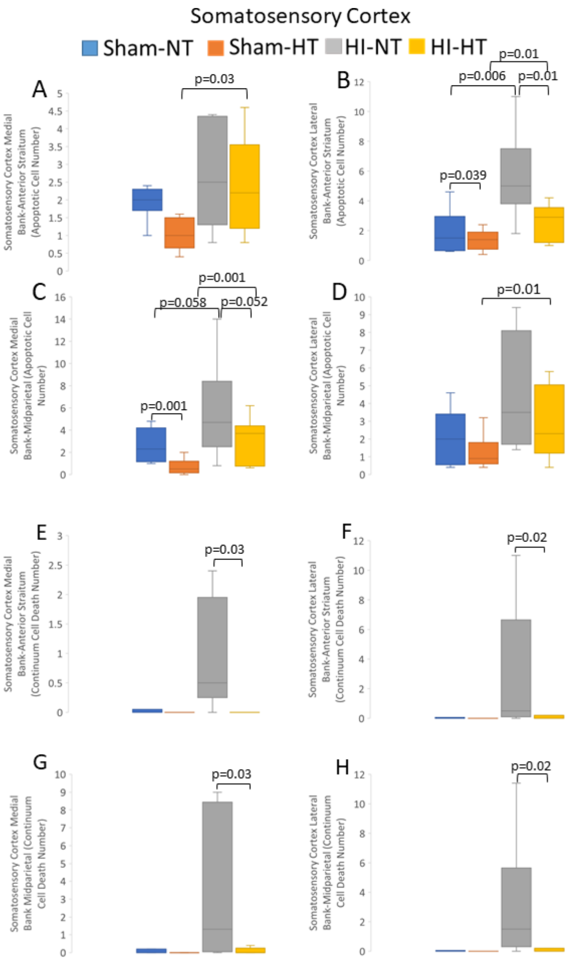

Supplementary Figure S6 Legend. Primary somatosensory cortex apoptosis and continuum cell degeneration in HI piglets with and without HT. **A,B.** Apoptotic cell number in the anterior somatosensory cortex at an anterior striatal level. **C,D.** Apoptotic cell number in the posterior somatosensory cortex at a midparietal/thalamus level. Compared to sham-NT piglets, HI-NT piglets had increased apoptosis in anterior somatosensory cortex later bank (*p*=0.006). HI-NT and HI-HT were not different. Sham-HT piglets had less apoptosis than did sham-NT piglets in anterior cortex lateral bank (p=0.039) and mid-parietal cortex medial bank (*p*=0.001). **E-H.** Apoptosis-necrosis continuum cell degeneration was significantly higher in HI-NT piglets compared to HI-HT in all parts of somatosensory cortex (*p*=0.02 or 0.03).

Supplementary Figure S7

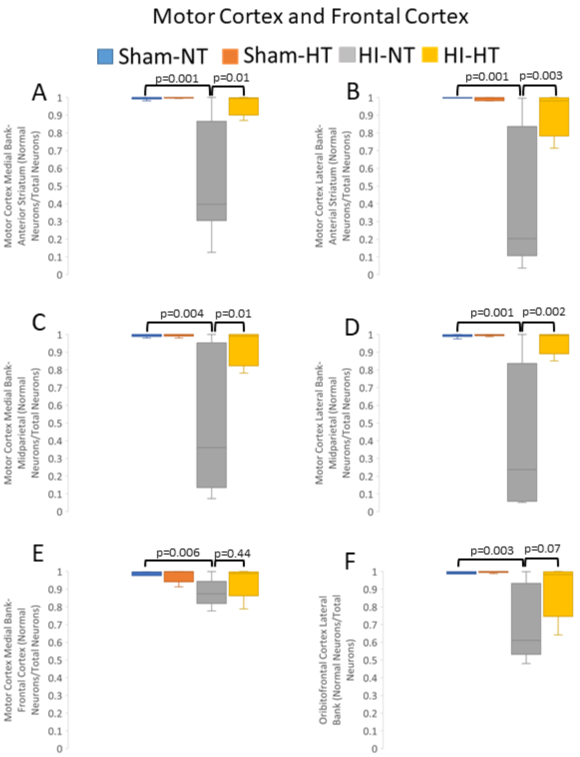

Supplementary Figure S7 Legend. Motor cortex damage after HI varied regionally in its anterior-posterior topography and was protected in HI-HT piglets: normal neuron-to-total neuron ratios. **A,B.** Motor cortex at an anterior striatal level. **C,D.** Posterior motor cortex at a midparietal/thalamus level. Compared to sham-NT piglets, HI-NT piglets had decreased normal-to-total neuron ratios in the motor cortex (anterior striatal level) medial bank (p=0.001) and lateral bank (p=0.001) and in the motor cortex (mid-parietal level) medial bank (p=0.004) and lateral bank (p=0.001). These motor cortical regions in HI-HT piglets had significant rescue from neuron loss compared to NI-NT. **E,F.** Prefrontal medial motor cortex and lateral orbitofrontal cortex. Compared to sham-NT piglets, HI-NT piglets had a significantly decreased normal-to-total neuron ratio in medial (p=0.006) and lateral (p=0.003) prefrontal cortex that was unmitigated in in HI-HT piglets.

Supplementary Figure S8

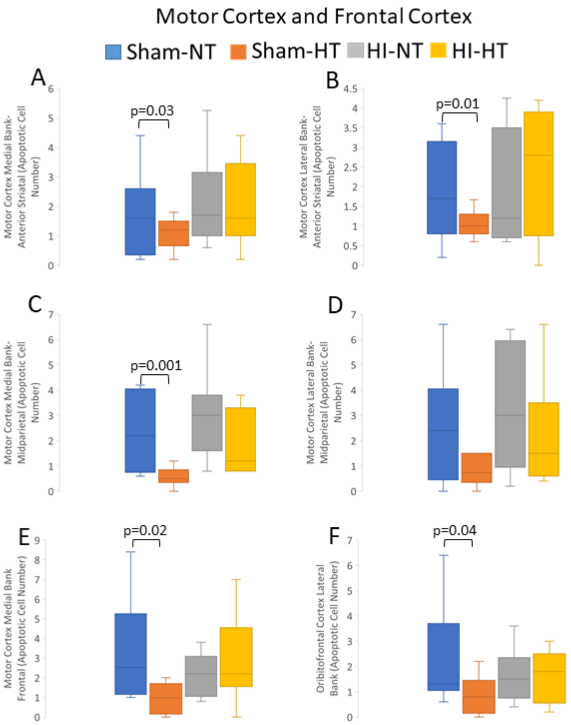

Supplementary Figure S8 Legend. Motor cortex apoptosis in sham and HI piglets with and without HT. Apoptotic cell number in the motor cortex (medial and lateral banks) at an anterior striatal level (**A,B**), at a midparietal/thalamus level (**C,D**), and at a prefrontal cortex level (**E,F**). Apoptosis was suppressed in most regions in sham HT piglets compared to sham-NT piglets.

Supplementary Figure S9

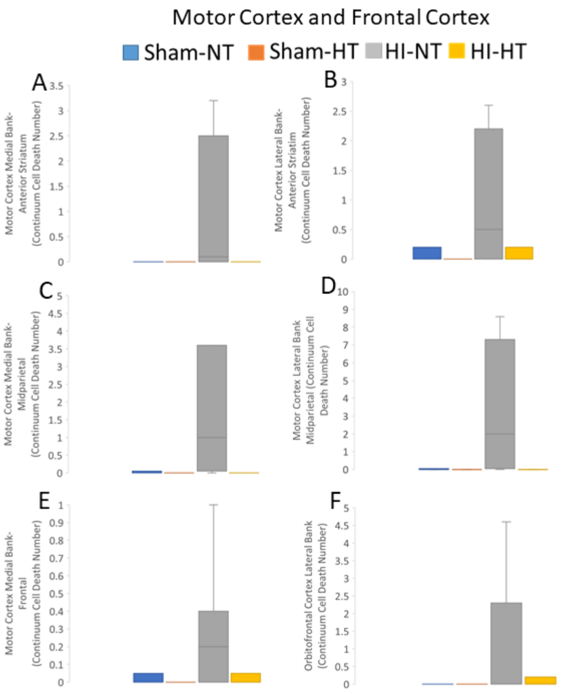

Supplementary Figure S9 Legend. Motor cortex continuum cell death in sham and HI piglets with and without HT. Apoptosis-necrosis continuum cell number in the motor cortex (medial and lateral banks) at an anterior striatal level (**A**,**B**), at a midparietal/thalamus level (**C**,**D**), and at a prefrontal cortex level (**E**,**F**). Continuum cell death did not differ significantly among piglet treatment groups.

Supplementary Figure S10 Legend. HT protects the putamen from HI injury

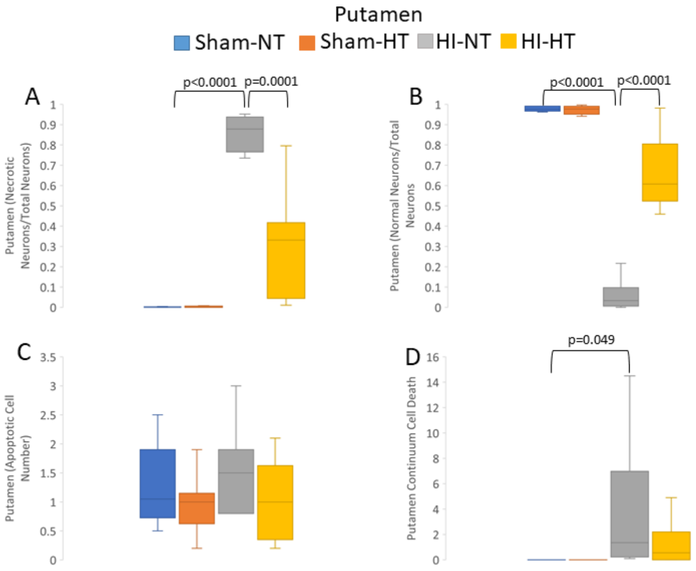

Supplementary Figure S10 Legend. HT protected neurons from HI injury in putamen. **A.** Neuronal ischemic necrosis was significantly greater in HI-NT piglets compared sham-NT piglets (p<0.0001) and HI-HT piglets (p=0.0001). **B.** Loss of normal neurons was significantly greater in HI-NT piglets compared sham-NT piglets (p<0.0001) and HI-HT piglets (p<0.0001) **C.** The number of cells with apoptosis did not differ among groups. **D.** The number of cells undergoing apoptosis-necrosis continuum cell death was greater (p=0.049) in HI-NT piglets compared to sham-NT piglets.

Supplementary Figure S11.

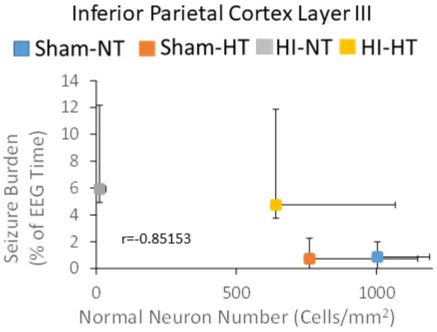

Supplementary Figure S11 Legend. Seizure burden and neuropathology in layer III of inferior parietal cortex. Group sizes are: Sham-NT (n=4), sham-HT (n=6), HI-NT (n=4), HI-HT (n=4). Values are means ± SD. Pearson correlation coefficient r. p=0.07.

**Supplementary Table S1. Cortical Region Comparisons of Ischemic-Necrotic/Total Neurons**		
Treatment Group Comparison	Cortical Region	*P* Value
Sham-NT vs HI-NT	Somatosensory Cortex, anterior striatal, medial bank	0.000604
	Somatosensory Cortex, anterior striatal, lateral bank	0.001441
	Somatosensory Cortex, midparietal, medial bank	0.001023
	Somatosensory Cortex, midparietal, lateral bank	0.001368
	Motor Cortex, anterior striatal, medial bank	0.004488
	Motor Cortex, anterior striatal, lateral bank	0.001285
	Motor Cortex, midparietal, medial bank	0.004421
	Motor Cortex, midparietal, lateral bank	0.001946
	Motor Cortex, frontal medial bank	0.004488
	Orbitofrontal Cortex, lateral bank	0.002305
Sham-HT vs HI-NT	Somatosensory Cortex, anterior striatal, medial bank	2.24889E-05
	Somatosensory Cortex, anterior striatal, lateral bank	7.98564E-05
	Somatosensory Cortex, midparietal, medial bank	4.86035E-05
	Somatosensory Cortex, midparietal, lateral bank	7.20889E-05
	Motor Cortex, anterior striatal, medial bank	0.000161
	Motor Cortex, anterior striatal, lateral bank	6.75572E-05
	Motor Cortex, midparietal, medial bank	0.000436
	Motor Cortex, midparietal, lateral bank	0.000128
	Motor Cortex, frontal medial bank	0.00318
	Orbitofrontal Cortex, lateral bank	0.000217656

Supplementary Table S2. Cortical Region Comparisons of Normal/ Total Neurons		
Treatment Group Comparison	Cortical Region	*P* Value
Sham-NT vs HI-NT	Somatosensory Cortex, anterior striatal, medial bank	0.000577
	Somatosensory Cortex, anterior striatal, lateral bank	0.001278
	Somatosensory Cortex, midparietal, medial bank	0.00054
	Somatosensory Cortex, midparietal, lateral bank	0.001073
	Motor Cortex, anterior striatal, medial bank	0.001651
	Motor Cortex, anterior striatal, lateral bank	0.001278
	Motor Cortex, midparietal, medial bank	0.004504
	Motor Cortex, midparietal, lateral bank	0.00142608
	Motor Cortex, frontal medial bank	0.005881
	Orbitofrontal Cortex, lateral bank	0.002088
Sham-HT vs HI-NT	Somatosensory Cortex, anterior striatal, medial bank	9.4518E-05
	Somatosensory Cortex, anterior striatal, lateral bank	8.72357E-05
	Somatosensory Cortex, midparietal, medial bank	1.71376E-05
	Somatosensory Cortex, midparietal, lateral bank	4.20856E-05
	Motor Cortex, anterior striatal, medial bank	9.4518E-05
	Motor Cortex, anterior striatal, lateral bank	8.72357E-05
	Motor Cortex, midparietal, medial bank	0.000431
	Motor Cortex, midparietal, lateral bank	7.58786E-05
	Motor Cortex, frontal medial bank	0.000660048
	Orbitofrontal Cortex, lateral bank	0.000208
